# Intracoronary infusion of Wharton’s jelly-derived mesenchymal stem cells in acute myocardial infarction: double-blind, randomized controlled trial

**DOI:** 10.1186/s12916-015-0399-z

**Published:** 2015-07-10

**Authors:** Lian R Gao, Yu Chen, Ning K Zhang, Xi L Yang, Hui L Liu, Zhi G Wang, Xiao Y Yan, Yu Wang, Zhi M Zhu, Tian C Li, Li H Wang, Hai Y Chen, Yun D Chen, Chao L Huang, Peng Qu, Chen Yao, Bin Wang, Guang H Chen, Zhong M Wang, Zhao Y Xu, Jing Bai, Di Lu, Yan H Shen, Feng Guo, Mu Y Liu, Yong Yang, Yan C Ding, Ye Yang, Hai T Tian, Qing A Ding, Li N Li, Xin C Yang, Xiang Hu

**Affiliations:** Center of Cardiology, Navy General Hospital, Beijing, China; Department of Cardiology, the First People’s Hospital of Foshan, Guangdong Province Foshan, China; Department of Cardiology, General Hospital of Armed Police Forces, Beijing, China; Shenzhen Beike Cell Engineering Research Institute, Guangdong Province Shenzhen, China; Department of Biostatistics, Peking University First Hospital, the Clinical Research Institute of Peking University, Beijing, China; Department of Cardiology, the General Hospital of Chinese People’s Liberation Army, Beijing, China; Department of Age Cardiology, the General Hospital of Chinese PLA, Beijing, China; Department of Cardiology, Beijing Huaxin Hospital, Beijing, China; Department of Cardiology, the Second Affiliated Hospital of Dalian Medical University, Liaoning Province Dalian, China; Department of Cardiology, the Central Hospital of Aerospace Corporation, Beijing, China; Department of Cardiology, General Hospital of Huabei Oilfield, Huabei Province Renqiu, China; Department of Cardiology, Beijing Chaoyang Hospital of Capital Medical University, Beijing, China; Department of Cardiology, Central Hospital of National Petroleum Corporation, Huabei Province Langfang, China; Department of Nuclear Medicine, Navy General Hospital, Beijing, China; Department of Ultrasonic Diagnosis, Navy General Hospital, Beijing, China

**Keywords:** Myocardial infarction, Mesenchymal stem cells, Wharton’s jelly of umbilical cord

## Abstract

**Background:**

The use of adult stem cells is limited by the quality and quantity of host stem cells. It has been demonstrated that Wharton’s jelly–derived mesenchymal stem cells (WJMSCs), a primitive stromal population, could integrate into ischemic cardiac tissues and significantly improve heart function. In this randomized, controlled trial, our aim was to assess the safety and efficacy of intracoronary WJMSCs in patients with ST-elevation acute myocardial infarction (AMI).

**Methods:**

In a multicenter trial, 116 patients with acute ST-elevation MI were randomly assigned to receive an intracoronary infusion of WJMSCs or placebo into the infarct artery at five to seven days after successful reperfusion therapy. The primary endpoint of safety: the incidence of adverse events (AEs) within 18 months, was monitored and quantified. The endpoint of efficacy: the absolute changes in myocardial viability and perfusion of the infarcted region from baseline to four months, global left ventricular ejection fraction (LVEF) from baseline to 18 months were measured using F-18-fluorodeoxyglucose positron emission computed tomography (F-18-FDG-PET) and 99mTc-sestamibi single-photon emission computed tomography (99mTc-SPECT), and two-dimensional echocardiography, respectively.

**Results:**

During 18 months follow-up, AEs rates and laboratory tests including tumor, immune, and hematologic indexes were not different between the two groups. The absolute increase in the myocardial viability (PET) and perfusion within the infarcted territory (SPECT) was significantly greater in the WJMSC group [6.9 ± 0.6 % (95 %CI, 5.7 to 8.2)] and [7.1 ± 0.8 % (95 %CI, 5.4 to 8.8) than in the placebo group [3.3 ± 0.7 % (95 %CI, 1.8 to 4.7), P <0.0001] and 3.9 ± 0.6(95 %CI, 2.8 to 5.0), P = 0.002] at four months. The absolute increase in the LVEF at 18 months in the WJMSC group was significantly greater than that in the placebo group [7.8 ± 0.9 (6.0 to approximately 9.7) vs. 2.8 ± 1.2 (0.4 to approximately 5.1), P = 0.001]. Concomitantly, the absolute decreases in LV end-systolic volumes and end-diastolic volumes at 18 months in the WJMSC group were significantly greater than those in the placebo group (P = 0.0004, P = 0.004, respectively).

**Conclusions:**

Intracoronary infusion of WJMSCs is safe and effective in patients with AMI, providing clinically relevant therapy within a favorable time window. This study encourages additional clinical trials to determine whether WJMSCs may serve as a novel alternative to BMSCs for cardiac stem cell-based therapy.

**Trial registration:**

Clinical Trials NCT01291329 (02/05/2011).

## Background

Coronary artery disease (CAD) remains a major world-wide cause of morbidity and mortality [1]. Despite recent advances in treatments for acute myocardial infarction (AMI), the irreversible loss of cardiomyocytes after an AMI leads to left ventricular (LV) remodeling and ischemic heart failure [1]. Regenerative cell therapies are emerging as potential treatments for AMI [2]. Studies have demonstrated the ability of transplanted mesenchymal stem cells (MSCs) to engraft, differentiate into cardiomyocyte-like and endothelial cells, and recruit endogenous cardiac stem cells [3–6]. Clinical evidence has shown that the intracoronary delivery of bone marrow mononuclear cells (BMMCs) or bone marrow mesenchymal stem cells (BMSCs) can improve the ejection fraction and reduce the infarct size [7–11]. However, the viability and function of autologous adult stem cells decline with age, especially in patients with MI [12, 13], which significantly limits their viability for clinical transplantation during the optimal window of opportunity to prevent adverse left ventricular remodeling [12]. Therefore, alternative sources of stem cells must be explored.

Wharton’s jelly-derived mesenchymal stem cells (WJMSCs), a primitive stromal population [14, 15], have been isolated from a continuum from the sub-amnion to the perivascular region of the umbilical cord [14, 15]. Wharton’s jelly of the umbilical cord originates from the extraembryonic and/or the embryonic mesoderm at day 13 of embryonic development [15]. WJMSCs retain a combination of most of their embryonic stem cell (ESC) and MSC markers in primary culture and early passages, thus retaining their multipotent stem cell characteristics [15–17]. Using Affymetrix GeneChip microarray and functional network analyses, we found for the first time that WJMSCs, except for their expression of stemness molecular markers in common with human ESCs (hESCs), exhibited a high expression of early cardiac transcription factor genes and could be induced to differentiate into cells expressing cardiac a-actin, troponin T and connexin-43 *in vitro* [18]. Moreover, growing evidence has shown that WJMSCs can be induced to differentiate into cardiomyocytes and endothelial cells and to integrate into the vasculature and ischemic cardiac tissue, as well as to improve heart function significantly [19–22].

In contrast to autologous adult stem cells, WJMSCs display greater cardiovascular differentiation potential [18–23], and more importantly, they are immune-privileged and can be transplanted into unrelated recipients [24]. This suggests the possibility of an allogeneic, “off-the-shelf” cell product, which can be used during the optimal time-frame for stem cell-based therapies after AMI, or even applied directly following revascularization of the AMI [15–17, 24]. WJMSCs constitute an attractive alternative to autologous MSCs for stem cell-based cardiac therapies [25].

Although our pilot clinical trial [26] and other clinical studies [10] using allogeneic stem cells showed promising results, there is no convincing evidence to date of the therapeutic safety and efficacy of the use of WJMSCs in humans. For this reason, this randomized, double-blind, multicenter trial was performed to investigate the therapeutic safety and efficacy of WJMSCs in patients with ST-elevation AMI.

## Methods

### Study population

Patients with ST-elevation AMI were admitted to cardiology centers in 11 hospitals in China between February 2011 and January 2012. A total of 160 subjects were enrolled in the trial. For inclusion in the study, patients had to satisfy the following eligibility criteria: 18- to 80-years old; a first ST-segment elevation MI; successful reperfusion with stent implantation of the infarct-related artery within 12 hours after the onset of symptoms; a substantial residual LV regional wall-motion abnormality (three or more hypokinetic LV segments observed on an echocardiograph after percutaneous coronary intervention, PCI); and creatine kinase (CK)-MB levels over three-fold the upper limit of the reference values. Exclusion criteria included previous Q-wave MI and severe coexisting conditions, such as advanced renal or hepatic dysfunction, and documented terminal illness or cancer. All subjects were administered medications according to the current updated ACC/AHA/SCAI guidelines along with standard rehabilitation programs for MI [27].

The study protocol conformed to the Declaration of Helsinki and was approved by the ethics committee of Navy General Hospital. All subjects signed written informed consent for enrollment in the study and treatment. The trial was monitored by an independent data and safety monitoring board (DSMB) who met every two months and as needed to assess adverse events.

### Study design and treatment randomization

**Fig. 1:**
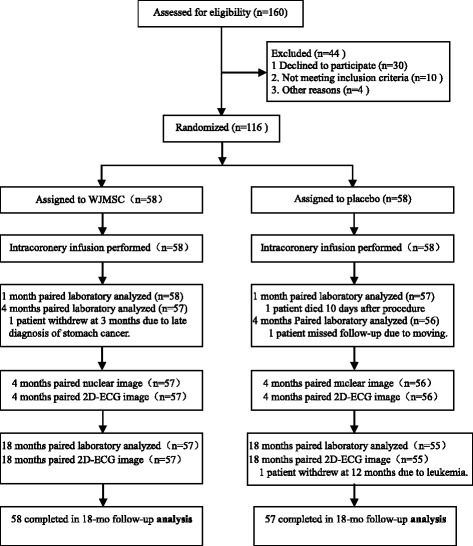
Enrollment and outcomes

The eligible patients were assigned randomly to each of two groups (WJMSCs or placebo control) in a 1:1 fashion using a computer-generated randomization of sequence numbers (Fig. 1). Physicians and other clinical personnel remained blind to the treatment assignment throughout the study. Day 0 was defined as the day of PCI. During days 5–7, all subjects were assigned randomly into either the WJMSC group, receiving 6 × 10^6^ WJMSCs through intracoronary infusion as described previously [28], or the placebo group, with a placebo injected via the same delivery method as that of the WJMSC group. Safety was evaluated on days 0 and 3, as well as 1, 4, 12 and 18 months post-treatment. Cardiac nuclear studies were performed pre-treatment and at four months post-treatment. Two-dimensional echocardiograms were measured before cell transfer and 4, 12 and 18 months after cell transplantation or placebo infusion.

### Study end points

The primary end point was safety: the incidence of adverse events (AEs) within 18 months, including death, nonfatal MI, stroke, hospitalization for worsening heart function, severe arrhythmias, repeated coronary intervention, stent thrombosis, coronary artery microvascular obstruction, immune system disorders, or ectopic tissue formation, was monitored and quantified. Laboratory assays, including biochemical assays, hematologic, tumor and immune indexes and Holter monitoring, were performed at the different follow-up times specified above.

The secondary end point was efficacy, which was assessed in terms of the absolute change in myocardial viability and perfusion in the infarcted region from the baseline to 4 months post-treatment, as well as the global LV ejection fraction (LVEF) from baseline to 18 months post-treatment, as measured by F-18-fluorodeoxyglucose positron emission computed tomography (F-18-FDG-PET), 99mTc-sestamibi single-photon emission computed tomography (99mTc-SPECT) and two-dimensional echocardiogram (ECG), respectively.

### Preparation and administration of WJMSCs

The protocol of WJMSCs preparation was approved by the General Logistics Department of the PLA and the Navy General Hospital Ethical Review Board. Twenty-one human umbilical cords were obtained, with the consent of the parents, from healthy donors, who had no complications throughout the pregnancy, no history of disease, and a full-term birth by caesarian section, and were aseptically stored in sterile saline and processed within six hours from partum to obtain the umbilical cord. After removal of blood vessels, the abundant extracellular matrix of Wharton’s jelly, which is a mucous tissue continuum from the subamnion to the perivascular region, was scraped off with a scalpel, finely cut and rinsed in sterile phosphate-buffered saline. The WJMSCs were isolated by a non-enzymatic method and cultured as described previously [18]. The WJMSCs were purified in a central cell-processing laboratory following the regulatory guidelines of the International Conference on Harmonization and the US Food and Drug Administration [29]. All procedures were performed and all solutions were prepared under Good Manufacturing Practice (GMP).

The infused WJMSCs were harvested at passage 3, during which ≥95 % of cells expressed CD29, CD73, CD90 and CD105, while the expression of CD45, CD34, CD14, CD79 and HLA-DR was 2 % or less. Released cells were negative for the pathogenic microorganisms HBV, HCV, HIV, cytomegalovirus, syphilis and exhibited ALT and endotoxin levels within 40 IU/L and 0.5 EU/mL, respectively. Final processing incorporated a total cell count and cell viability (≥85 %) determination by trypan blue testing. Based on the results from our animal experiments, we decided on a dose of 6 × 10^6^ WJMSCs by intracoronary transplantation in this trial. In brief, a dose-escalation study for intracoronary delivery of WJMSCs involved 12 pig models of AMI, weighing 28–35 kg, of mixed gender. The dose was escalated at 1, 2, 3, 6 x 10^6^ with a 30-minute interval. A coronary angiogram as well as left ventriculogram was obtained at 15 minutes following each infusion. Blood flow to the distal left anterior descending artery (LAD), measured under fluoroscopy by counting the number of heart beats required to fill this region of the vessel with contrast, was not changed at bolus doses up to 6 × 10^6^ WJMSCs. Significant changes in LV wall motion were revealed until administration of the 3 × 10^6^ dose [30]. The placebo consisted of a vehicle (saline with 10,000 U/L heparin) injected without cells.

The cells were shipped at 4 °C and delivered to each catheterization laboratory at the 11 participating cardiology centers, using a standard operating procedure. After extensive discussion with the enrolled subjects, written informed consent was obtained before initiating treatment. After arterial puncture and administration of 8 000 U heparin, 6 × 10^6^ WJMSCs dispersed in 10 mL heparinized saline (saline with 10,000 U/L heparin), or the placebo, were infused using a stop-flow technique through an over-the-wire balloon catheter positioned within the stent segment as described previously [28]. Contrast medium was injected into the infarct-related artery to ascertain vessel patency after cell infusion.

### F-18-FDG-PET and 99mTc-sestamibi SPECT examination

F-18-FDG- PET (GE Millennum VG Hawkeye, Israel) and 99mTc-sestamibi SPECT (Varicam, GE-Elscint, Haifa, Israel) was performed in patients at one day before and four months after the procedure. After an overnight fast for at least 12 hours, patients were given an oral glucose load according to their serum glucose level. Sequential measurements of serum glucose were made until the serum glucose level reached 7.8 – 8.9 mmol/L and then 18F-FDG (222–296 MBq) and 99mTc-sestamibi (555–740 MBq) were injected intravenously, respectively. Myocardial images were reconstructed using a standard filtered back projection and displayed as a series of short-axis, horizontal and vertical long-axis slices. Mean signal intensities were measured in the respective areas supplied by the three major coronary arteries in three-axis views. Results were calculated using F-18–FDG-PET and 99mTc-sestamibi SPECT bull’s-eye views. All parameters were analyzed independently by two experienced observers who were unaware of the treatment assignment.

### Two-dimensional echocardiogram examination

Patients who received cell grafts or standard treatment underwent echocardiography consecutively 1–2 days before cell/placebo infusion and at 4, 12 and 18 months follow-up. A 17-segment echocardiogram was performed to measure regional left ventricular wall motion score, end-systolic volume, end-diastolic volume and LVEF by using standard methods of the American Society of Echocardiography [31], and were analyzed independently by two experienced observers who were unaware of patients’ treatment assignments.

### Statistical analyses

The population size of patient enrollment was influenced by the difficulty in selecting eligible patients because the inclusion criteria used in our study selected only those patients with AMI who had undergone primary PCI within 12 hours and who had also agreed to accept a WJMSC intracoronary transplant five days after their first PCI operation. Very few patients were deemed eligible. To address this issue, we expanded the study to include more hospitals. We also performed a statistical analysis specifically to determine whether the study population was normally distributed. To ascertain the efficacy, including PET and SPECT at month 4, and two dimensional-ECG at months 4, 12 and 18, with variables adjusted by baseline values, analysis of covariance (ANCOVA) was performed to assess differences between the placebo and WJMSC treatment groups. To estimate the treatment effect, differences in least-squares means and the corresponding 95 % confidence intervals (CI) were calculated based on the ANCOVA model. The Wilcoxon or Student’s t-test was used to compare changes between the baseline and follow-up values according to the distribution of variables. Categorical variables were analyzed by chi-square or Fisher’s exact test, as appropriate. Continuous variables were expressed as means ± standard errors (SE) unless otherwise stated. Categorical data were presented as frequencies and percentages. All statistical tests were two-sided, and P <0.05 was considered statistically significant. All analyses were performed using SAS software, version 9.3 (SAS Institute, Cary, NC, USA).

## Results

### Participant characteristics

A total of 160 subjects with ST-elevation AMI were screened, and 116 eligible subjects signed written informed consent to participate in the study. All patients had second generation drug eluting stents inserted in primary PCI (Table 1). After five days, they were assigned evenly and randomly to the WJMSC or placebo groups (Fig. 1). The baseline characteristics and clinical interventions of reperfusion and medication were well-matched in the two groups (Table 1). During the 18-month follow-up period in the placebo group, one patient withdrew for missed follow-up due to a move, one patient died 10 days after discharge and one patient could not complete the follow-up at 12 months due to a diagnosis of leukemia. In the WJMSC group, one subject could not complete the four month follow-up due to a diagnosis of advanced stomach cancer (Fig. 1). Therefore, the findings from 58 patients in the WJMSC group and 57 patients in the placebo group were analyzed statistically in this trial as shown in Fig. 1.

**Table 1:** Baseline characteristics of the patients

Characteristic	Placebo	WJMSCs	P value
	(n = 58)	(n = 58)	
**Risk factor**			
Age (years)	56.7±1.7	57.3±1.3	0.79
Men - number (%)	51(87.9)	55(94.8)	0.18
Body-mass index (kg/m^2^)	25.4±0.3	24.9±0.3	0.26
Diabetes mellitus - number (%)	14(24.1)	17(29.3)	0.52
Hyperlipidemia - number (%)	22(37.9)	21(36.2)	0.84
Hypertension - number (%)	26(44.8)	33(56.9)	0.19
Smoking (current of former) -number (%)	32(55.2)	34(58.6)	0.70
Family history of coronary heart disease - number (%)	20(34.5)	17(29.3)	0.55
**Coronary artery disease - number (%)**			0.19
1-Vessel disease	28(48.3)	21(36.2)	
2-Vessel disease	16(27.6)	14(24.1)	
3-Vessel disease	14(24.1)	23(39.7)	
**Infarct treatment**			
Infarct-related artery - number (%)			0.55
Left anterior descending artery	31(53.4)	29(50.0)	
Left circumflex artery	6(10.3)	10(17.2)	
Right coronary artery	21(36.2)	19(32.8)	
PCI for additional stenoses in non-infarct-related vessels - number (%)	10(17.2)	15(25.9)	0.25
Time from symptom onset to first reperfusion therapy-hour			
Mean	7.3±0.5	7.2±0.5	0.81
Median	6.3	7.3	
Drug-eluting stent - number (%)	58(100)	58(100)	1
Gp II b/IIIa inhibitor during acute PCI - number (%)	7(12.1)	8(13.8)	0.78
Intravenous catecholamines - number (%)	3(5.2)	4(6.9)	0.69
Maximal creatine kinase (U/L)	1899±273	1653±206	0.93
Maximal creatine kinase MB (U/L)	192±21	170±17	0.42
Maximal troponin T (μg/L)	18.2±3.1	24.7±4.1	0.61
**TIMI flow grade**			
Before PCI - number (%)			0.93
Grade 0 or 1	42(72.4)	42(72.4)	
Grade 2	8(13.8)	7(12.1)	
Grade 3	8(13.8)	9(15.5)	
After PCI - number (%)			0.55
Grade 0 or 1	0(0)	0(0)	
Grade 2	1(1.7)	2(3.4)	
Grade 3	57(98.3)	56(96.6)	
**Baseline quantitative measure of LV function**			
Global left ventricular ejection fraction (%)			
Mean	51.1±1.0	52.0±0.9	0.51
Left venticular fractional shortening (%)			
Mean	26.4±0.6	27.2±0.6	0.33
Wall motion score index (17-segment model)			
Mean	1.28±0.03	1.29±0.02	0.62
End-systolic volume (ml)			
Mean	64.6±2.8	63.3±2.7	0.68
End-diastolic volume (ml)			
Mean	129.9±3.5	130.4±3.6	0.90
**Cell therapy**			
Time from reperfusion to infusion of study therapy-days			
Mean	6.3±0.1	6.1±0.1	0.25
Median	6.5	6.0	
TIMI flow grade before study therapy			
Mean	2.98±0.02	2.97±0.02	0.56
Median	3	3	
TIMI flow grade after study therapy			
Mean	2.98±0.02	2.97±0.02	0.56
Median	3	3	
**Current medication - number (%)**			
Aspirin and clopidogrel	58(100)	58(100)	1
Beta-blocker	48(82.8)	42(72.4)	0.18
ACE-inhibitors or angiotensin-receptor B	43(74.1)	42(72.4)	0.83
Stains	54(93.1)	54(93.1)	1

Data are means (SE) or number (%) unless otherwise stated. TIMI trial grades are defined as follows: grade 0, no perfusion; grade 1, penetration without perfusion; grade 2, partial perfusion; and grade 3, complete perfusion. *ACE* angiotensin-converting enzymes, *LV* left ventricular, *PCI* percutaneous coronary intervention

### Adverse events (AEs)

The major adverse cardiac events (MACEs) and other clinical AEs encountered are summarized in Table 2. No major peri-procedural complications occurred in either group. As mentioned above, one subject in the placebo group suffered sudden cardiac death 10 days after discharge. In the WJMSC group, one subject was re-hospitalized due to heart failure induced by a respiratory infection two weeks post-treatment, and one subject required re-vascularization for in-stent restenosis in the infarct-related coronary artery four months post-treatment. No new arrhythmias were recorded on the Holter monitor study during the 18-month follow-up. The groups did not differ in occurrences of MACEs, including death, recurrences of AMIs and re-hospitalization due to heart failure, during the course of treatment and the 18-month follow-up period.

**Table 2:** Clinical events during the 18 months follow-up period

Event	Placebo	WJMSCs	P value
**Events during procedure**	(n=58)	(n=58)	
Death	0	0	
Obstruction of related-vessel	0	0	
Severe arrhythmia	0	0	
**Events 6-mo follow-up (cumulative)** ^**a**^	(n=57)	(n=58)	
Death	1	0	0.49^b^
Recurrence MI	0	0	
Rehospitalization for heart failure	0	1	1.0^b^
Stent thrombosis	0	0	
Revascularization	0	1	1.0^b^
Cerebral infarction	0	0	
Arrhythmia	0	0	
Immune system disorder			
Ectopic tissue formation	0	1	1.0^b^
**Combined events**			
Death or MI	1	0	0.49^b^
Death, recurrence of MI, revascularization procedure	1	1	1^b^
Death, MI, or rehospitalization for heart failure	1	1	1^b^
Death, MI, stroke, rehospitalization for heart failure, severe arrhythmia	1	1	1^b^
Immune system disorder, ectopic tissue formation	0	1	1^b^
**Events 12-mo follow-up (cumulative)**			
Death	1	0	0.49^b^
Recurrence MI	0	0	
Rehospitalization for heart failure	0	1	1.0^b^
Stent thrombosis	0	0	
Revascularization	0	1	1.0^b^
Cerebral infarction	0	0	
Arrhythmia	0	0	
Immune system disorder	0	0	
Ectopic tissue formation	1	1	1.0^b^
**Combined events**			
Death or MI	1	0	0.49^b^
Death, recurrence of MI, revascularization procedure	1	1	1^b^
Death, MI, or rehospitalization for heart failure	1	1	1^b^
Death, MI, stroke, rehospitalization for heart failure, severe arrhythmia	1	1	1^b^
Immune system disorder, ectopic tissue formation	1	1	1^b^
**Events 18-mo follow-up (cumulative)**			
Death	1	0	0.49^b^
Recurrence MI	0	0	
Rehospitalization for heart failure	0	1	1.0^b^
Stent thrombosis	0	0	
Revascularization	0	1	1.0^b^
Cerebral infarction	0	0	
Arrhythmia	0	0	
Immune system disorder	0	0	
Ectopic tissue formation	1	1	1.0^b^
**Combined events**			
Death or MI	1	0	0.49^b^
Death, recurrence of MI, revascularization procedure	1	1	1^b^
Death, MI, or rehospitalization for heart failure	1	1	1^b^
Death, MI, stroke, rehospitalization for heart failure, severe arrhythmia	1	1	1^b^
Immune system disorder, ectopic tissue formation	1	1	1^b^

^*a*^1 Patient was lost in the placebo group. ^b^Fisher’s exact test was used

*MI* myocardial infarction

In the WJMSC group, one patient was diagnosed with advanced stomach cancer three months post-treatment. However, no evidence of ectopic tissue formation and no increase in the levels of tumor-associated antigens were observed in the remaining subjects during the follow-up. WJMSC infusion induced neither acute nor persistent immune or biochemical abnormalities, as shown in Table 3.

**Table 3:** Quantitative measures of blood index

Blood index	Placebo	WJMSCs	P value
	(number = 55)	(number = 57)	
**CD3(%)**			
Baseline	66.8±0.9	67.6±0.8	0.48
3 Days	67.6±1.1	69.1±0.9	0.29
P value(baseline vs.3d)	0.43	0.08	
1 Mo	67.3±1.0	66.6±0.9	0.53
P value (baseline vs.1 mo)	0.58	0.18	
4 Mo	65.2±0.7	66.3±0.9	0.34
P value (baseline vs.6 mo)	0.06	0.11	
12 Mo	66.2±0.9	66.4±0.8	0.81
P value (baseline vs.12 mo)	0.64	0.16	
18 Mo	67.1±1.0	66.8±0.9	0.76
P value (baseline vs.18 mo)	0.66	0.29	
**CD4(%)**			
Baseline	39.6±1.2	40.1±0.8	0.70
3 Days	40.4±1.1	39.3±0.8	0.39
P value (baseline vs.3 d)	0.37	0.24	
1 Mo	38.2±1.0	37.1±0.7	0.34
P value (baseline vs.1 mo)	0.09	<0.0001	
4 Mo	37.7±1.1	38.0±0.8	0.75
P value (baseline vs.6 mo)	0.09	0.02	
12 Mo	37.6±0.9	38.2±0.6	0.58
P value (baseline vs.12 mo)	0.11	0.03	
18 Mo	38.8±1.1	39.2±0.9	0.45
P value (baseline vs.18 mo)	0.09	0.16	
**CD8(%)**			
Baseline	21.9±0.9	22.4±0.8	0.39
3 Days	21.3±0.8	22.9±0.8	0.13
P value (baseline vs.3 d)	0.36	0.16	
1 Mo	21.5±0.5	22.8±0.7	0.14
P value (baseline vs.1 mo)	0.63	0.47	
4 Mo	21.5±0.7	21.8±0.7	0.78
P value (baseline vs.6 mo)	0.66	0.32	
12 Mo	23.2±0.4	22.2±0.6	0.16
P value (baseline vs.12 mo)	0.12	0.80	
18 Mo	22.4±0.7	23.6±0.9	0.21
P value (baseline vs.18 mo)	0.56	0.71	
**IgG(g/L)**			
Baseline	11.1±0.3	10.7±0.3	0.42
3 Days	11.3±0.3	11.0±0.2	0.34
P value (baseline vs.3d)	0.41	0.39	
1 Mo	11.5±0.4	10.9±0.4	0.13
P value (baseline vs.1 mo)	0.10	0.63	
4 Mo	10.9±0.4	10.3±0.4	0.48
P value (baseline vs.6 mo)	0.74	0.36	
12 Mo	11.8±0.3	11.3±0.4	0.31
P value (baseline vs.12 mo)	0.09	0.13	
18 Mo	11.6±0.5	11.1±0.5	0.18
P value (baseline vs.18 mo)	0.11	0.64	
**IgM(g/L)**			
Baseline	1.10±0.10	1.07±0.08	0.80
3 Days	1.15±0.09	1.05±0.07	0.38
P value (baseline vs.3d)	0.24	0.67	
1 Mo	1.24±0.11	1.23±0.10	0.69
P value (baseline vs.1 mo)	0.28	0.15	
4 Mo	1.17±0.10	1.05±0.08	0.35
P value (baseline vs.6 mo)	0.50	0.69	
12 Mo	1.14±0.06	1.15±0.05	0.55
P value (baseline vs.12 mo)	0.66	0.19	
18 Mo	1.35±0.11	1.31±0.10	0.59
P value (baseline vs.18 mo)	0.39	0.24	
**CEA(ng/ml)**			
Baseline	2.21±0.14	2.63±0.17	0.08
3 Days	2.47±0.15	2.55±0.17	0.71
P value (baseline vs.3 d)	0.03	0.48	
1 Mo	2.33±0.14	2.76±0.19	0.11
P value (baseline vs.1 mo)	0.33	0.40	
4 Mo	2.37±0.15	2.42±0.15	0.91
P value (baseline vs.6 mo)	0.25	0.21	
12 Mo	2.33±0.18	2.44±0.16	0.62
P value (baseline vs.12 mo)	0.58	0.34	
18 Mo	2.22±0.16	2.55±0.24	0.16
P value (baseline vs.18 mo)	0.21	0.34	
**CRP(mg/L)**			
Baseline	5.72±0.53	6.47±0.49	0.27
3 Days	4.99±0.51	5.47±0.48	0.31
P value (baseline vs.3 d)	0.12	0.06	
1 Mo	3.70±0.40	3.54±0.31	0.80
P value (baseline vs.1 mo)	<0.0001	<0.0001	
4 Mo	3.20±0.33	3.34±0.30	0.54
P value (baseline vs.6 mo)	<0.0001	<0.0001	
12 Mo	3.04±0.19	3.00±0.29	0.46
P value (baseline vs.12 mo)	<0.0001	<0.0001	
18 Mo	3.56±0.43	3.42±0.38	0.76
P value (baseline vs.18 mo)	<0.0001	<0.0001	
**ALT(U/L)**			
Baseline	30.5±1.8	32.8±2.1	0.41
3 Days	31.6±2.1	32.9±2.7	0.85
P value (baseline vs.3 d)	0.42	0.98	
1 Mo	30.3±1.8	28.3±1.3	0.68
P value (baseline vs.1 mo)	0.96	0.47	
4 Mo	29.3±1.1	29.9±1.2	0.72
P value (baseline vs.6 mo)	0.87	0.59	
12 Mo	27.2±1.1	27.4±1.1	0.94
P value (baseline vs.12 mo)	0.36	0.31	
18 Mo	29.2±1.4	28.1±1.2	0.61
P value (baseline vs.18 mo)	0.83	0.43	
**TBIL(μmol/L)**			
Baseline	16.7±1.5	15.1±1.3	0.63
3 Days	11.6±0.6	11.5±0.5	0.89
P value (baseline vs.3 d)	0.0003	0.006	
1 Mo	15.1±0.7	13.8±0.6	0.32
P value (baseline vs.1 mo)	0.32	0.31	
4 Mo	14.5±1.0	16.0±1.1	0.27
P value (baseline vs.6 mo)	0.22	0.60	
12 Mo	14.2±0.7	15.6±0.5	0.12
P value (baseline vs.12 mo)	0.14	0.76	
18 Mo	14.6±0.7	13.4±0.6	0.36
P value (baseline vs.18 mo)	0.31	0.28	
**BUN(mmol/L)**			
Baseline	5.30±0.18	5.63±0.22	0.28
3 Days	4.91±0.19	5.19±0.17	0.12
P value (baseline vs.3 d)	0.06	0.07	
1 Mo	5.10±0.18	5.26±0.23	0.96
P value (baseline vs.1 mo)	0.36	0.22	
4 Mo	5.29±0.17	5.21±0.18	0.47
P value (baseline vs.6 mo)	0.95	0.13	
12 Mo	5.42±0.18	5.46±0.20	0.89
P value (baseline vs.12 mo)	0.68	0.57	
18 Mo	5.22±0.23	5.33±0.32	0.85
P value (baseline vs.18 mo)	0.46	0.29	
**CRE(μmol/L)**			
Baseline	84.5±2.3	81.8±2.2	0.39
3 Days	81.2±2.0	77.9±2.1	0.26
P value (baseline vs.3 d)	0.14	0.14	
1 Mo	78.5±2.4	76.3±2.0	0.46
P value (baseline vs.1 mo)	0.06	0.07	
4 Mo	83.5±2.2	78.2±2.0	0.07
P value (baseline vs.6 mo)	0.71	0.17	
12 Mo	84.0±2.0	80.4±1.8	0.19
P value (baseline vs.12 mo)	0.84	0.63	
18 Mo	82.6±2.1	79.7±2.7	0.48
P value (baseline vs.18 mo)	0.56	0.61	

Plus–minus values are means ± SE. P values of between-group comparisons were determined by Student’s t-test or nonparametric Mann–Whitney U tests. P values of within-group comparisons were determined by ANOVA with 95 % CIs. *ALT* alanine aminotransferase, *ANOVA* analysis of variance, *BUN* blood urea nitrogen, *CEA* carcino-embryonic antigen, *CI* confidence interval, *CRP* C reactive protein, *TBIL* total bilirubin

### Myocardial viability

**Fig. 2:**
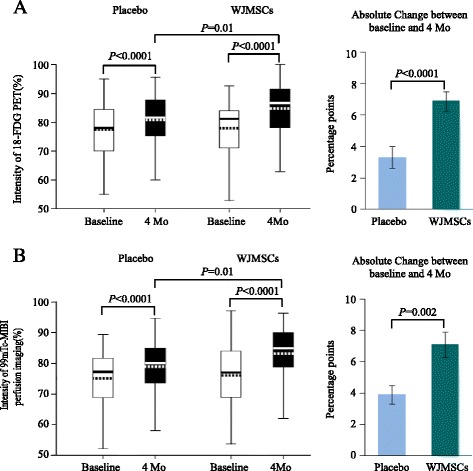
**a** Mean F-18-FDG-PET signal intensity in the infarct vessel area measured before and four months after transplantation. Comparison of mean F-18-FDG-PET signal intensity changes and absolute changes between the WJMSC group and the placebo group using analysis of covariance (ANCOVA). T-bars show the standard errors. **b** Mean signal intensity of 99mTc-MIBI perfusion imaging in the infarcted vessel area measured before and four months after transplantation. Comparison of mean 99mTc-MIBI signal intensity changes and absolute changes between the WJMSC group and the placebo group by ANCOVA. T-bars show the standard errors *WJMSC* Wharton’s jelly-derived mesenchymal stem cells

No difference in the intensity of F-18-FDG-PET signals was observed between the WJMSC and placebo group at baseline. However, there was a significantly greater increase in the tracer uptake within the infarcted area from baseline to four months post-treatment in the WJMSC group compared with the placebo group. As shown in Fig. 2a, the absolute increase in the F-18-FDG-PET signal intensity was markedly higher in the WJMSC group (6.9 ± 0.6 %, 95 % CI 5.7−8.2) than in the placebo group (3.3 ± 0.7 %, 95 % CI 1.8−4.7), (P<0.0001).

### Myocardial perfusion

The 99mTc-SPECT imaging analysis of myocardial perfusion showed a similar total infarcted area at baseline in both groups (Fig. 2b). An increase in myocardial perfusion was observed in both groups four months post-treatment compared with baseline (Fig. 2b). However, as shown in Fig. 2b, there was a significantly higher absolute 99mTc-SPECT signal intensity in the WJMSC group (7.1 ± 0.8 %, 95 % CI 5.4−8.8) compared with the placebo group (3.9 ± 0.6, 95 % CI 2.8−5.0), (P = 0.002).

### Left ventricular function

**Fig. 3:**
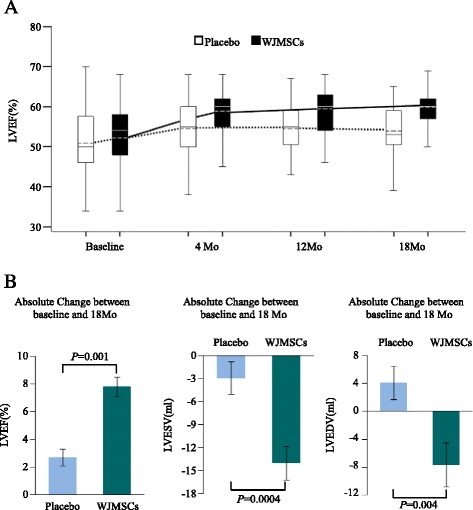
**a** Comparison of changes in the global left ventricular ejection fraction (LVEF) between the WJMSC group and the placebo group measured by two dimensional-echocardiograph before transplantation and at the 4-, 12- and 18-month follow-ups. **b**. The absolute changes in LVEF, LVESV and LVEDV between the WJMSC group and the placebo group at 18 months measured by ECG and analyzed by ANCOVA. T-bars show the standard errors *ANCOVA* analysis of covariance, *ECG* echocardiogram, *LVEDV* left ventricular end-diastolic volume, *LVESV* left ventricular end-systolic volume, *WJMSC* Wharton’s jelly-derived mesenchymal stem cells

As shown in Fig. 3 and Table 4, patients treated with WJMSCs experienced a 7.8 ± 0.9 (6.0 to approximately 9.7) LVEF increase over baseline at 18 months compared with the 2.8 ± 1.2 (0.4 to approximately 5.1) increase in the placebo group (p = 0.001). Concomitantly, the absolute decreases in LV end-systolic volumes and LV end-diastolic volumes at 18 months in the WJMSC group were significantly greater than those in the placebo group (P = 0.0004, P = 0.004, respectively).

**Table 4:** Quantitative measures of left ventricular function

Variable	Placebo (95 % CI)	WJMSCs(95 % CI)	P value
	(number = 55)	(number = 57)	
**Global LVEF (%)**			
Baseline			
Mean	51.0±1.1(48.8~53.3)	52.1±1.0(50.2~54.1)	0.46
4 Months			
Mean	54.2±0.9(52.5~56.1)	58.5±0.7(57.1~60.0)	0.0003
Difference	3.2±0.9(1.4~5.0)	6.4±0.7(4.9~7.9)	0.008
P value (baseline vs.6 months)	0.0009	<0.0001	
12 Months			
Mean	54.5±0.8(52.8~56.2)	58.8±0.8(57.3~60.4)	0.0002
Difference	3.5±0.8(1.9~5.0)	6.7±0.8(5.1~8.3)	0.004
P value (baseline vs.12 months)	<0.0001	<0.0001	
18 Months			
Mean	54.0±0.8( 52.3~55.7)	60.0±0.5 (58.9~61.0)	<0.0001
Difference	2.8±1.2(0.4~5.1)	7.8±0.9(6.0~9.7)	0.001
P value (baseline vs.18 months)	0.05	<0.0001	
**LVFS (%)**			
Baseline			
Mean	26.6±0.7(25.2~27.9)	27.3±0.6(26.0~28.6)	0.42
4 Months			
Mean	29.7±0.7(28.3~31.0)	32.6±0.6(31.4~33.9)	0.002
Difference	3.1±0.6(1.9~4.3)	5.3±0.6(4.0~6.6)	0.01
P value (baseline vs.6 months)	<0.0001	<0.0001	
12 Months			
Mean	29.8±0.6(28.5~31.0)	32.7±0.5(31.7~33.8)	0.0005
Difference	3.2±0.5(2.1~4.3)	5.4±0.5(4.3~6.5)	0.005
P value (baseline vs.12 months)	<0.0001	<0.0001	
18 Months			
Mean	28.6±0.6(20.8~35.6)	33.6±0.5(27.3~39.1)	<0.0001
Difference	2.1±0.8(−8.9~13.5)	6.2±0.7(−4.1~16.4)	<0.0001
P value (baseline vs.18 months)	0.01	<0.0001	
**WMSI (17-segment model)**			
Baseline			
Mean	1.28±0.03(1.23~1.34)	1.29±0.03(1.24~1.34)	0.76
4 Months			
Mean	1.16±0.02(1.11~1.21)	1.10±0.01(1.07~1.12)	0.02
Difference	−0.12±0.02(−0.16~−0.09)	−0.19±0.02(−0.23~−0.16)	0.003
P value (baseline vs.6 months)	<0.0001	<0.0001	
12 Months			
Mean	1.15±0.02(1.11~1.20)	1.09±0.02(1.06~1.12)	0.02
Difference	−0.13±0.02( −0.17~−0.10)	−0.20±0.02(−0.23~−0.17)	0.003
P value (baseline vs.12 months)	<0.0001	<0.0001	
18 Months			
Mean	1.16±0.9(1.02~1.40)	1.07±0.01(1.00~1.17)	<0.0001
Difference	−0.13±0.02(−0.42~0.08)	−0.22±0.02(−0.57~0.04)	0.01
P value (baseline vs.18 months)	<0.0001	<0.0001	
**End-systolic volume (ml)**			
Baseline			
Mean	66.0±3.1(59.8~72.2)	63.1±2.8(57.5~68.7)	0.38
4 Months			
Mean	63.4±2.8(57.7~69.0)	55.9±2.1(51.7~60.2)	0.07
Difference	−2.6±1.8(−6.4~1.1)	−7.2±1.9(−10.9~−3.5)	0.09
P value (baseline vs.6 months)	0.16	0.0003	
12 Months			
Mean	62.5±2.8(56.9~68.0)	55.1±2.1(50.9~59.4)	0.06
Difference	−3.5±1.9(−7.3~0.2)	−8.0±2.0(−12.1~−3.9)	0.12
P value (baseline vs.12 months)	0.11	0.0002	
18 Months			
Mean	62.8±0.9(59.1~66.6)	49.2±1.0(47.1~51.2)	<0.0001
Difference	−2. 9±2.1(−7.1~1.3)	−14.0±2.2(−18.4~−9.5)	0.0004
P value (baseline vs.18 months)	0.2	<0.0001	
**End-diastolic volume (ml)**			
Baseline			
Mean	132.2±3.9(124.3~140.0)	130.3±3.8(122.7~137.9)	0.49
4 Months			
Mean	136.0±4.0(127.9~144.0)	133.2±3.2(126.8~139.5)	0.72
Difference	3.8±2.3(−0.7~8.4)	2.9±2.6(−2.3~8.0)	0.78
P value (baseline vs.6 months)	0.20	0.07	
12 Months			
Mean	134.5±3.9(126.6~142.4)	131.9±3.1(125.8~138.1)	0.60
Difference	2.4±2.5(−2.6~7.3)	1.6±2.8(−4.0~7.2)	0.84
P value (baseline vs.12 months)	0.45	0.22	
18 Months			
Mean	136.4±3.1(130.1~142.7)	122.7±1.6 (119.5~125.8)	0.0001
Difference	4.1±2.4(−0.8~9.0)	−7.6±3.13(−13.9~−1.4)	0.004
P value (baseline vs.18 months)	0.1	<0.0001	

LVEF, LVESV and LVEDV by two dimensional-ECG from baseline to 18 months post-treatment between the WJMSC group and placebo-control group were analyzed by ANCOVA. Treatment effects are expressed as differences in least-squares means (ANCOVA model) with 95 % CI *ANCOVA* analysis of covariance, *CI* confidence interval, *ECG* echocardiogram, *LVEDV* left ventricular end-diastolic volume, *LVESF* left ventricular end-systolic volume, *WJMSC* Wharton’s jelly-derived mesenchymal stem cells

## Discussion

In this randomized, double-blind, controlled trial, we reported the safety and efficacy of intracoronary allogeneic WJMSC transplantation in patients with ST-elevation AMI. A reduction in myocardial infarct size and improved heart function were confirmed by the myocardial viability and perfusion measurements, as well as the global LVEF. Importantly, WJMSC transplantation prevented post-infarct LV adverse remodeling, as evidenced by the changes in the LVEDV and LVESV at 18 months.

After a heart attack, an optimal window of opportunity exists during which stem cell-based therapies can exert a therapeutic effect [7]. Thus, stem cells that meet clinical grade parameters in terms of purity, potency, identity, and dose must be readily available. However, the desired dose is difficult to obtain with autologous MSCs, because they cannot be expanded and processed in less than 14 days [12, 13], thus reducing their usefulness due to missing the optimal time window for cell treatment after AMI. This study provides an exploratory roadmap for the introduction of a promising and urgently needed cellular therapy in clinical practice, based on the concurrent microenvironmental conditions and stage of the disease. It promises an off-the-shelf product for AMI patients, backed by a solid procedure and a validated infrastructure that can be administered to eligible patients during the most favorable time window.

Notably, no signs of an immune response triggered by the allogeneic WJMSC transplantation, ectopic tissue formation or increased levels of tumor-associated antigens were observed in this study. One patient in the WJMSC group was diagnosed with advanced stomach cancer at the three-month follow-up; however, the tumor-associated antigen findings and pathological examination results indicated that the tumor was not likely to have derived from the WJMSCs. In addition, the intracoronary injection of WJMSCs, unlike BMSCs which could be associated with acute complications such as coronary occlusion during transfer surgery [12], did not impair the TIMI flow grade or trigger an increase in troponin concentrations, demonstrating that no microvascular obstruction or coronary artery occlusion had occurred during or after the treatment (Table 2). Taken together, the current results support the conclusion that WJMSC transplantation is relatively safe at least up to 18 months.

Based on previous studies and meta analyses [7–11], there were slight but significant improvements (from 2.5 to 3.66 % of LVEF) in LV function and a significant decrease in the infarct size −4.03 % of patients with AMI after autologous bone marrow stem cell therapy. Cardiac regeneration is defined as regrowth of lost or destroyed cardiomyocytes. Here, we verified that the transplanted WJMSCs significantly increased myocardial viability within the infarcted area as measured by F-18-FDG-PET, which is considered sensitive and specific for assessing viable myocardium [32, 33]. Concomitantly, the myocardial perfusion in the infarcted area and the LVEF were increased significantly after WJMSC transplantation. Notably, even though the infarct size (−6.9 %) was not reduced greatly after the transfer of the WJMSCs, the gradual improvements in LVEDV and LVESV over 18 months in the WJMSC group demonstrated that these cells could prevent adverse LV remodeling effectively. Previous studies have shown that the transfer of BMMCs had no significant impact on LVEDV, suggesting that BMMCs may have a limited effect on LV remodeling after AMI [7, 8]. Taken together, our findings indicate that combined optimal reperfusion therapy (stent implantation) and intracoronary administration of WJMSCs during the optimal time frame can decrease the infarct size, enhance the recovery of global and regional LV function and prevent LV remodeling after AMI.

Our study was not designed to assess the underlying mechanisms of WJMSC treatment that promote functional recovery after AMI. Nevertheless, evidence supports the idea that WJMSCs constitute a unique cell family with a high degree of stemness and unique transcriptional profiles [34, 35]. We previously reported higher expression levels of mesoderm and specialized cardiac progenitor cell genes, including Brachyury (T), mesoderm posterior 1, Flk-1, Nkx2.5 and Isl-1, which represent key transcription factors of cardiac development [18]. These key characteristics of WJMSCs indicate their strong potential to differentiate into cardiomyocytes, endothelia, and to form neovascular networks, to integrate into ischemic cardiac tissues, all of which result in the improvement of heart function [18–22]. Moreover, WJMSCs can secrete large amounts of anti-apoptotic, angiogenic factors and growth factors, exerting paracrine effects for the regeneration of myocardium and coronary vessels [36].

Tumorigenesis is a major concern in the clinical application of human ESCs and induced pluripotent stem cells (iPS). Studies have demonstrated that normal karyotypes were observed in WJMSCs harvested from primary, early and late passages, and teratomas were not induced after WJMSC injection into severe combined immune deficient (SCID) mice [37]. No ectopic tissues or increased levels of tumor-associated antigens were observed after WJMSC transplantation in this trial. Furthermore, WJMSCs can regulate immunity by modulating the behavior of natural killer (NK) cells and T-cell populations to evade immune responses and are well-tolerated in allogeneic transplantation [24, 26].

In this study, a relatively lower dose of WJMSCs was administered for intracoronary infusion because in our previous clinical trial one patient suffered from a serious complication of coronary artery occlusion during the BMSC injection procedure [12]. However, the wide safety margin found in the present study indicated that higher doses of WJMSCs would be safe for clinical trials in the future.

There is a limitation to this proof-of-concept study. Although we used an especially important tool, PET, for interpreting myocardial viability and predicting improvements in cardiac function, contrast-enhanced magnetic resonance imaging (CE-MRI) was not available in all centers to assess cardiac function. This may be a drawback compared to recently related publications.

## Conclusions

We demonstrated for the first time that intracoronary delivery of prepared clinical-grade WJMSCs, a true stem cell population with highly expressing early cardiac transcription factors, was safe in treating patients with an AMI attack and could significantly improve myocardial viability and heart function. Our original study provides an exploratory roadmap to translate the promising cellular therapy into clinical practice according to the urgency for treatment, the concurrent microenvironmental conditions and the stage of disease. Therefore, this study encourages additional clinical trials to determine whether WJMSCs may serve as a novel allogeneic source for cardiac stem cell-based therapies.

## Abbreviations

99mTc-SPECT: 99mTc-sestamibi single-photon emission computed tomography
AEs: adverse events
AMI: acute myocardial infarction
F-18-FDG-PET: F-18-fluorodeoxyglucose positron emission computed tomography
LVEF: Left ventricular ejection fraction
WJMSC: Wharton’s jelly–derived mesenchymal stem cell
